# Focus On: Epigenetics and Fetal Alcohol Spectrum Disorders

**Published:** 2011

**Authors:** Michael S. Kobor, Joanne Weinberg

**Keywords:** Maternal alcohol consumption, prenatal alcohol exposure, fetal alcohol spectrum disorders, birth defects, teratogenesis, gastrulation, preconception, conception, pregnancy, genetics, epigenetics

## Abstract

Epigenetic changes—stable but potentially reversible alterations in a cell’s genetic information that result in changes in gene expression but do not involve changes in the underlying DNA sequence—may mediate some of the detrimental effects of prenatal alcohol exposure and contribute to the deficits and abnormalities associated with fetal alcohol spectrum disorders. These epigenetic processes are linked to the chromatin (i.e., DNA, histone proteins, and other associated proteins) and commonly involve chemical modifications (e.g., methylation) of these molecules, which may result in altered expression of the affected genes. Even alcohol exposure prior to conception appears to be able to induce epigenetic changes in the parental genetic material that can be passed on to the offspring and affect offspring outcome. Similarly, epigenetic processes may occur as a result of maternal alcohol consumption during the period between fertilization of the egg and implantation in the uterus. The period most sensitive to alcohol’s adverse effects appears to be gastrulation, which corresponds to prenatal weeks 3 to 8 in the human and prenatal days 7 to 14 in the mouse, when cells are differentiating to form organs. One way in which alcohol exposure may induce epigenetic changes, particularly abnormal DNA methylation, is by affecting a set of biochemical reactions called the methionine–homocysteine cycle.

Children born to women who consume alcohol during pregnancy may exhibit a range of abnormalities and developmental deficits that together are termed fetal alcohol spectrum disorders (FASD) (Manning and Hoyme 2007; Stratton et al. 1996). Fetal alcohol syndrome (FAS), which can occur with chronic consumption of high doses of alcohol, represents the most severe end of the spectrum (Jones et al. 1973). Consumption of alcohol at levels that do not produce full FAS can result in more limited deficits that are classified under different categories. These include the following (Stratton et al. 1996):

- Partial FAS, where only some of the growth or central nervous system (CNS) deficits or characteristic facial features are present;
- Alcohol-related birth defects (ARBD), which primarily are characterized by physical abnormalities; or
- Alcohol-related neurodevelopmental disorder (ARND), which primarily is characterized by neurobehavioral deficits.

Although the timing and level of alcohol exposure clearly affect the type and severity of the resulting effects, the specific alcohol-induced deficits observed and their extent can vary quite substantially, even among individuals with similar levels of exposure. It appears that multiple direct and indirect mechanisms, likely activated at different stages of development or at different dose thresholds of exposure, may contribute to this varying pattern of deficits (Goodlett et al. 2005). Furthermore, increasing evidence indicates that both genetic and epigenetic mechanisms may play a role in mediating the broad range of effects reported in children with FASD. After providing a brief overview of some of the genetic mechanisms contributing to FASD, this article will take a closer look at the epigenetic mechanisms that may underlie alcohol’s detrimental effect during prenatal development.

## Genetic Mechanisms

Several lines of evidence cumulatively point to a role for genetic variables that may contribute to the diverse adverse outcomes related to alcohol exposure in utero (for reviews, see Green et al. 2007; Warren and Li 2005). Several promising approaches have been developed to elucidate genetic involvement in the susceptibility to or protection from alcohol-induced prenatal toxicity and birth defects (i.e., teratogenesis). Three areas of investigation will be discussed briefly here.

One line of research has analyzed variations in the DNA sequence (i.e., polymorphisms) of genes involved in alcohol metabolism (Jacobson et al. 2006; McCarver et al. 1997; Stoler et al. 2002; Viljoen et al. 2001). Much of this research has focused on polymorphisms in the genes encoding the enzyme alcohol dehydrogenase (ADH), which catalyzes the oxidation of ethanol to acetaldehyde. Several ADH-encoding genes have been identified, and each of these may exist in more than one variant (i.e., allele). Some studies specifically have examined the *ADH1B* gene. Although the results have been somewhat mixed depending on the population studied, the level of maternal drinking in pregnancy, and age at which the children were tested, data suggest that the presence of either of two variants of this gene in the mother—ADH1B*2 and ADH1B*3—may provide protection from adverse effects of alcohol exposure (Jacobson et al. 2006; Warren and Li 2005). For example, Jacobson and colleagues (2006) found that drinking at conception was less frequent in the presence of the maternal ADH1B*3 allele, and virtually no adverse effects were found in children whose mothers had at least one ADH1B*3 allele. Interestingly, there was no consistent pattern of greater vulnerability in children lacking the ADH1B*3 allele. The mechanisms by which protection through the maternal genotype occurs have not yet been determined. Other studies have sought to identify genes that are potential targets of alcohol’s teratogenic effects and thus are important in determining susceptibility to FASD (Lombard et al. 2007). Through this approach, researchers have identified several genes that are involved in various signalling pathways playing critical roles in embryogenesis and development. These genes now are being studied further to determine their possible role in mediating alcohol’s adverse effects on the fetus. Finally, a third line of studies has used two closely related C57BL/6 mouse lines, B6J and B6N, to elucidate differences in genetic susceptibility of the developing face and brain to alcohol-induced abnormalities (Green et al. 2007). These analyses, combined with other ongoing studies on target genes, should prove useful in future work aimed at elucidating mechanisms underlying alcohol teratogenesis.

## Epigenetic Mechanisms

In addition to these genetic studies, investigation of possible epigenetic mechanisms as mediators of alcohol’s adverse effects on the fetus provides another promising approach for understanding the complex observable characteristics (i.e., phenotypes) associated with FASD and the persistence of these characteristics into adulthood (Haycock 2009). The term epigenetics refers to stable, but potentially reversible, alterations in a cell’s genetic information that result in changes in gene expression but do not involve changes in the underlying DNA sequence (i.e., mutations). Emerging evidence suggests that the creation of epigenetic changes at certain sites (i.e., loci) in the genome depends, at least in part, on environmental cues. Thus, epigenetic mechanisms may function as mediators connecting the genome to environmental signals and exposures (e.g., in utero alcohol exposure). These epigenetic changes can persist long after the transient environmental signal has disappeared, highlighting the particular importance of this phenomenon for FASD.

Perhaps the best understood epigenetic mechanism involves the regulation of gene expression—that is, the process through which the genetic information encoded in the DNA directs the production of RNA and proteins. In general, epigenetic modifications serve to establish and maintain different gene expression programs in different cell types. Differential gene expression ensures that the more than 200 different cell types that make up mammalian tissues differ in phenotype or appearance, even though almost all cells in an organism essentially share the same genetic information. Epigenetic mechanisms contribute, at least in part, to this differential gene expression.

**Figure 1:**
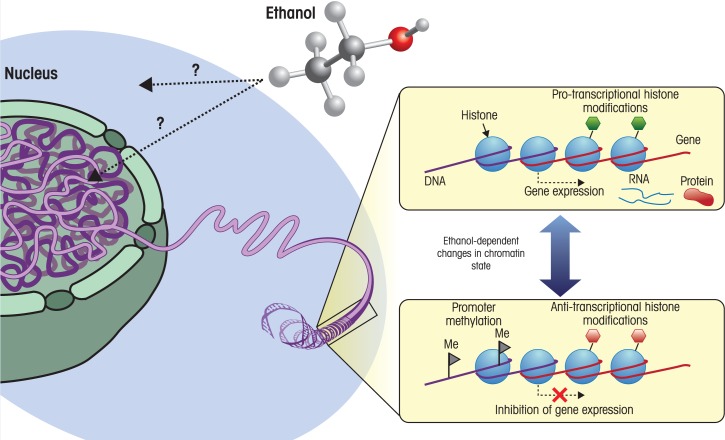
Environmental factors can cause changes in chromatin state. Chromatin, which is comprised primarily of DNA spooled around histone proteins, is located in the cell nucleus. This DNA-packaging system is dynamic, and the local state of chromatin influences gene expression—the processes by which the information encoded in genes is used to create RNA (i.e., transcription) and, ultimately, proteins. The addition of methyl groups (i.e., methylation) to certain DNA building blocks (i.e., cytosine nucleotides) in a regulatory region of a gene called the promoter tends to inhibit transcription of that gene. Histone proteins also can be modified by the addition of various molecules, which influences the recruitment and binding to chromatin of various complexes involved in transcription. These modifications can either promote transcription (i.e., are protranscriptional) or have an inhibitory effect on transcription (i.e., are antitranscriptional), depending on the nature of the modification and the state of the surrounding chromatin. Both DNA methylation and histone modification are dynamic processes, with numerous enzymes that remove and replace these modifications. The prevalence and activity of these enzymes, as well as the availability of substrate (e.g., methyl groups), can be influenced by environmental factors acting at the cellular and nuclear level. For example, ethanol may disrupt the homocysteine-methionine cycle, resulting in a decrease in the levels of S-adenosyl methionine, which acts as a methyl donor for DNA and histone methylation.

Epigenetic processes are tightly linked to the structure of chromatin—the complex of DNA, histone proteins, and additional associated proteins that forms the chromosomes—and involve modifications of both the DNA and the histone proteins (see figure 1). For example, one well-studied component of epigenetic regulation of gene expression is chemical modification of the DNA itself by a reaction called methylation. Methylation involves the addition of methyl (CH_3_) groups to cytosine, which is one of the four bases that make up DNA.^1^ This methylation occurs primarily in the context of DNA sequences where a cytosine nucleotide occurs next to a guanine nucleotide, separated by a phosphate group (i.e., CpG dinucleotides). If cytosine methylation occurs in the DNA region called the promoter region of a gene, which directs the first step in the production of RNA and protein from DNA information (i.e., transcription) or in another regulatory region of a gene, it is sensed by proteins that turn gene expression on or off. In most cases, these proteins then recruit other proteins or enzymes that, in turn, participate in epigenetic modifications of histone proteins, including chemical modifications such as acetylation, methylation, phosphorylation, and other processes.

In concert, the components of the epigenetic machinery serve to regulate gene activity, often in a stable manner. Because of this chemical stability, as well as the relative ease of appropriate analytic methods, DNA methylation is the most accessible epigenetic mark and, consequently, has most often been used in population-based and other human epigenetic studies to date. Moreover, given the extensive crosstalk between different chromatin marks, DNA methylation also can serve as an indicator for additional alterations, such as histone modifications at a given gene promoter.

### Alcohol Intake and Epigenetic Modifications in Adults

**Figure 2:**
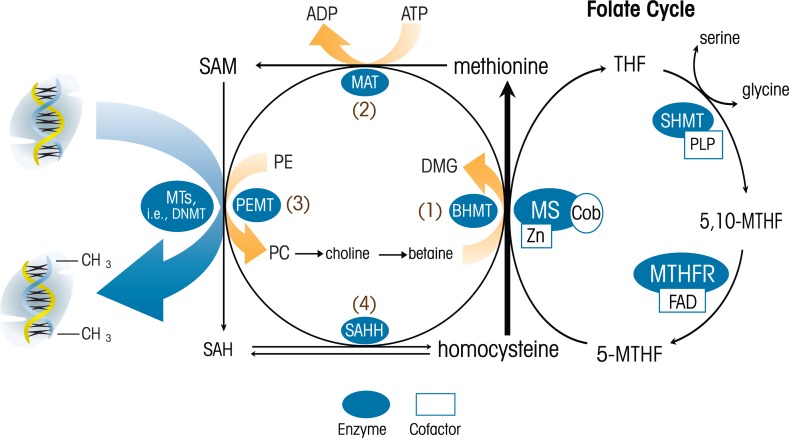
Schematic of the methionine-homocysteine cycle in the liver. Methionine is an essential amino acid that plays critical roles in cell functions such as protein synthesis. It also is a precursor to S-adenosyl methionine (SAM), which serves as the methyl donor for numerous methylation reactions. These reactions, which are mediated by enzymes called methyl transferases (MTs), involve the transfer of methyl groups from a donor (e.g., SAM) to a recipient (e.g., DNA or a protein). This methylation results in altered function or activity of the recipient molecule. The demethylated SAM then is further processed to homocysteine, which in turn is converted back into methionine either through a folate-dependent mechanism (i.e., the folate cycle on the right side of the figure) or a folate-independent mechanism (in the center of the figure). NOTES: 5-MTHF = 5-methyltetrahydrofolate; 5,10-MTHF = 5,10-methylenetetrahydrofolate; BHMT = betaine:homocysteine methyltransferase; Cob = cobalamin; DHF = dihydrofolate; DMG = dimethylglycine; FAD = flavin adenine dinucleotide; MAT = methionine adenosyltransferase; MTHFR = methylenetetrahydrofolate reductase; MS = methionine synthase; MTRR = methionine synthase reductase; MTs = methyl transferases; PLP = pyridoxal phosphate; SAH = S-adenosylhomocysteine; SAHH = SAH hydrolase; SAM = S-adenosylmethionine; THF = tetrahydrofolate; Zn = zinc.

Persuasive evidence from studies of adult alcoholics and animal models of alcoholism points to multiple connections between alcohol and the epigenome. For example, alcohol has been shown to disrupt a set of reactions called the methionine–homocysteine cycle at multiple points (Barak et al. 1987; Finkelstein et al. 1974; Halsted et al. 1996). Methionine is an essential amino acid—that is, it cannot be synthesized by the body and must be taken in from the diet. It has a critical role in normal cell function, including protein synthesis, and is a precursor of the compound *S*-adenosyl methionine (SAM). In the methionine-homocysteine cycle, methionine is activated to SAM, ultimately resulting in the formation of homocysteine. Methionine then is regenerated through folate-dependent or folate-independent methylation of homocysteine. Homocysteine also can enter other pathways through which it ultimately is excreted (see figure 2). SAM serves as a key methyl donor for numerous reactions, including DNA and histone methylation. Alcohol can affect the methionine–homocysteine cycle by disrupting the enzymes required for methionine metabolism (Barak et al. 1993; Lieber 1988; Lu et al. 2000), thus interfering with SAM-dependent methylation reactions. Indeed, global changes in tissue methylation capacity and DNA methylation now are considered key characteristics of alcoholic liver disease (Mato and Lu 2007; Shukla et al. 2008).

Alcohol and its breakdown products (i.e., metabolites such as acetaldehyde) also cause different site-specific modifications in histones (Shukla et al. 2008), as shown both in primary cultures of rat liver cells (Park et al. 2003) and in rats acutely treated with alcohol in vivo (Kim and Shukla 2006). Evidence suggests that ingestion of alcohol and other drugs may sensitize the liver such that it is predisposed to exaggerated injury when exposed to toxic compounds later in life. Changes in the acetylation and methylation patterns of specific histones resulting from chronic alcohol feeding and high blood alcohol levels can lead to persistently altered gene expression and thus may play a role in this “epigenetic memory.”

Alcohol additionally may influence epigenetic regulatory mechanisms through several other pathways. For example, alcohol may reduce the absorption of folate, a key component of the methionine-homocysteine cycle (Halsted et al. 2002; Naughton et al. 1989), thereby influencing methylation capacity. Furthermore, consistent with the hypothesis that alcohol induces modification of the epigenome at specific loci, analyses of white blood cells (i.e., peripheral blood lymphocytes) in humans with alcohol dependence demonstrated abnormal DNA methylation patterns in the promoter regions of several genes involved in various aspects of development (Bonsch et al. 2006; Hillmacher et al. 2008). One might hypothesize that patient lymphocyte profiles can serve as a sentinel of epigenetic patterns existing in the brain, the tissue most salient for the etiology of FASD. However, this hypothesis can be validated and probed in detail only in animal models. This issue is a prime example of how research with human populations can drive research utilizing animal models. For example, loci with altered DNA methylation patterns identified in human studies could be interrogated in lymphocytes from laboratory animals to test for physiological conservation. DNA methylation patterns of conserved loci in lymphocytes then can be measured in distinct brain regions salient to FASD pathology. Importantly, work with animal models can, in turn, inform studies with patient populations. Based on findings in the animal work, for example, imaging techniques could be used in patient populations to study the major brain regions identified.

## Prenatal Alcohol Exposure and Epigenetics

Investigation of the role of epigenetic mechanisms in the adverse effects of prenatal alcohol exposure in both humans and animal models still is at an early stage, although its potential role is becoming more widely recognized (Haycock 2009). During prenatal and early postnatal development, the epigenome is highly susceptible to environmental stimuli such as diet, drugs, environmental agents, and maternal behavior. Three developmental periods—preconception, preimplantation, and gastrulation—appear to be particularly sensitive to the teratogenic effects of alcohol, and it has been suggested that the clinical variability of FASD may be related not only to the dose of alcohol but also to when alcohol exposure occurs in relation to these developmental periods (Haycock 2009; Haycock and Ramsay 2009). Importantly, these three periods also are peak periods of epigenetic reprogramming. Of these periods, gastrulation appears to be most sensitive to teratogenic insults (Armant and Saunders 1996), and alcohol exposure at this time induces a wide range of adverse effects, including morphological and behavioral abnormalities. However, the finding that both preconception and preimplantation alcohol exposure can cause adverse effects, even though the embryo is not directly exposed to alcohol at this stage because it is not yet implanted in the uterus and therefore not connected to or nourished by the maternal system, probably provides the most compelling evidence of a role for epigenetic mechanisms in the outcomes observed.

### Effects of Preconception Alcohol Exposure

Some of the earliest evidence of a preconception effect of alcohol comes from a study by Stockard (1913) showing that if normal female rats are mated with male rats that had been chronically exposed to alcohol,^2^ the offspring exhibited greatly increased perinatal mortality; moreover, these effects persisted even into the next generation. More recent studies support these findings of preconception effects mediated by paternal alcohol consumption. For example, reduced birth weight and length, reduced litter size, and increased malformations in the offspring have been reported (Abel 2004; Anderson et al. 1981; Mankes et al. 1982), although fetal vulnerability to alcohol-induced decreases in body weight was shown to have a maternal genetic contribution as well (Sittig and Redei 2010). One potential mechanism for how paternal alcohol consumption might induce these effects is suggested by the finding that expression of a key enzyme catalyzing DNA methylation—called DNA methyltransferase 1 (DNMT1)—was reduced in the sperm of paternal rats after 9 weeks of alcohol exposure (Bielawski et al. 2002). Similarly, analysis of methylation patterns of sperm DNA from human volunteers showed a correlation between chronic alcohol use and demethylation of DNA regions that normally show particularly high methylation (i.e., are hypermethylated) (Ouko et al. 2009). Transmission of these epigenetic changes to the offspring through fertilization possibly could alter gene expression in the fetus, thus affecting prenatal development.

Preconception effects of maternal alcohol consumption also have been reported, including effects on birth weight and growth (Livy et al. 2004). This observation is supported by a recent study by Kaminen-Ahola and colleagues (2010), who studied offspring of female mice that had been given free access to an alcohol solution for 10 weeks prior to conception. The analysis focused on a specific allele of a gene called *Agouti viable yellow (A^vy^*). This allele has been called an epigenetic biosensor for environmental effects on the fetus (Waterland 2006), because prenatal exposure to nutritional and toxic agents can affect *A^vy^* expression in the offspring through epigenetic regulation. Specifically, lower-than-normal DNA methylation (i.e., DNA hypomethylation) of a promoter element for *A^vy^* is associated with constitutive expression of the *Agouti* gene, which is reflected phenotypically in a yellow coat color. Conversely, hypermethylation correlates with blunted expression (i.e., silencing) of the *Agouti* gene, which in turn causes a brownish pseudo-agouti coat color. Intermediate methylation and expression of the *Agouti* gene results in mottled coat color. Importantly, these differences in gene expression and coat color can occur in genetically identical animals, suggesting that it is a purely epigenetic effect.

In their study, Kaminen-Ahola and colleagues (2010) found that in offspring of alcohol-exposed female mice, the probability of transcriptional silencing of the allele was increased. Thus, preconception alcohol exposure more than doubled the percentage of pseudo-agouti offspring compared with that in the control group, consistent with a higher probability of hypermethylation and decreased expression of *A^vy^*. However, these effects likely are only indirect, because the relevant *A^vy^* allele in the offspring was inherited from the fathers and was not present in the unfertilized eggs of the alcohol-consuming mothers.

### Effects of Preimplantation Alcohol Exposure

The preimplantation period—that is, the time after the egg cell has been fertilized and before it implants in the uterus—encompasses the first 4 to 6 days of mouse and rat development, which corresponds roughly to the first 2 weeks of human pregnancy. Very few studies have examined the teratogenic effects of in vivo alcohol exposure during this period. One study (Padmanabhan and Hameed 1988) using administration of high alcohol doses (5.8 g/kg) directly into the abdominal cavity of the mothers during the preimplantation period found marked adverse outcomes on gestation day 15, including malformations and growth retardation. A recent study by Haycock and Ramsay (2009) investigated the effects of genomic imprinting on alcohol-induced fetal growth retardation following preimplantation exposure. Imprinting is a classical epigenetic phenomenon that involves expression of specific genes primarily from the alleles inherited from one of the parents rather than from the alleles inherited from both parents. The investigators found that although placentas and embryos of alcohol-exposed mothers showed severe growth retardation compared with those of control animals, alcohol exposure did not alter overall DNA methylation in the embryos. However, the alleles inherited from the fathers were significantly less methylated in the placentas of alcohol-exposed animals compared with the placentas from control animals, suggesting effects on imprinting as a possible novel mechanism underlying alcohol-induced growth retardation. In contrast, several studies using alcohol exposure of rat embryos in culture (i.e., in vitro exposure) found no adverse effects on preimplantation embryos, suggesting that alcohol’s teratogenic effects during this developmental period may depend on an interaction with the maternal system (for a review, see Haycock 2009).

### Effects of Alcohol Exposure During Gastrulation

Gastrulation is the stage early during embryonic development that follows implantation of the embryo into the uterine wall, which in mice is completed by day 6. Gastrulation encompasses the onset of organ development, which occurs on days 7 to 14 of gestation in the mouse and during weeks 3 to 8 in humans. During this period, extensive cellular differentiation occurs and the various organs begin to form. As mentioned above, the gastrulation period generally is considered the most sensitive to teratogenic insult because differentiating cells appear to be particularly vulnerable to the teratogenic effects of alcohol.

An early study by Garro and colleagues (1991) was one of the first to suggest that epigenetic alterations may occur following alcohol exposure during gastrulation. In this study, acute alcohol administration to pregnant female mice on gestation days 9 to 11 resulted in global hypomethylation of fetal DNA. More detailed analysis suggested that this hypomethylation likely resulted from a direct inhibitory effect of alcohol on DNMT activity. Because changes in the pattern of DNA methylation in many cases might be related to changes in gene expression, such alcohol-induced hypomethylation might alter gene expression programs that could contribute to the developmental abnormalities seen in FASD. However, the effects of alcohol on DNA methylation and gene expression most likely are more nuanced, as evidenced by the discussion above regarding hypermethylation of the fetal *A^vy^* locus caused by maternal alcohol exposure (Kaminen-Ahola et al. 2010).

Further support for the role of epigenetic mechanisms during gastrulation comes from a recent study by Liu and colleagues (2009), who cultured whole mouse embryos and exposed them to a high dose of alcohol^3^ for 44 hours, beginning at embryonic day 8.25. Overall, the investigators observed delayed and reduced growth, as well as numerous abnormalities, including alterations in the heart, the caudal neural tube (i.e., tail end of the developing central nervous system), brain vesicles, optic system, and limb buds. Moreover, there was a more than 10-fold increase in the number of genes with increased promoter DNA methylation on chromosomes 10 and X in alcohol-exposed embryos with a neural tube defect compared with embryos without a neural tube defect. Significant changes in methylation also were seen in the promoter regions of genes known to be involved in the cell cycle, growth, programmed cell death (i.e., apoptosis), cancer, and sense of smell (i.e., olfaction). Consistent with the concept of epigenetic regulation of gene expression, alterations in DNA methylation were associated with significant changes in the expression of 84 genes.

Interestingly, effects of maternal alcohol consumption on the *Agouti A^vy^* allele were found not only during the preimplantation period, as discussed above, but also during the early gastrulation period, illustrating the utility of the epigenetically sensitive *A^vy^* allele as an environmental reporter in FASD research (Kaminen-Ahola et al. 2010). In these studies, female mice were exposed to an ethanol solution for 8 days after fertilization, and, as described above, alterations in *A^vy^* expression were evaluated by coat color of the offspring. Consistent with the outcome observed following preconception exposure, alcohol exposure during gastrulation resulted in a significantly higher proportion of pseudo-agouti offspring compared with that in the control group. These data suggest that alcohol can influence *A^vy^* expression early in development by increasing the probability of transcriptional silencing at this particular locus, mediated by increased DNA methylation. In addition, the study provided evidence for *A^vy^*-independent persistent alterations in global gene expression profiles and detected phenotypic manifestations reminiscent of FASD. This finding strongly suggests a broad role for epigenetic mechanisms in the etiology of FASD (Kaminen-Ahola et al. 2010).

A recent study by Haley and colleagues (2006) that evaluated the stress response in 5- to 7-month-old human infants also provides support for effects of alcohol exposure during the gastrulation period. The mothers in this study reported significant reductions in alcohol consumption after learning of their pregnancies. Nevertheless, the mean percent of prenatal drinking days from conception to pregnancy recognition (which corresponds roughly to the preimplantation period and extends a few weeks into gastrulation) was related to increases in reactivity of the stress hormone cortisol, elevated heart rate, and increased negative affect in their infants in response to a social-emotional stressor.^4^ These effects were statistically significant after controlling for the effects of maternal depression and annual income. Although not discussed by Haley and colleagues (2006), the finding that alcohol caused marked effects in 5- to 7-month-old infants, even though exposure occurred very early in gestation and for only a brief period, suggests the possible influence of an epigenetic mechanism. In other words, transient exposure to alcohol during the preimplantation and early gastrulation periods of development may have permanently altered gene expression patterns in basic cell-signaling pathways involved in limbic/neuroendocrine development, resulting in reprogramming of the hypothalamic-pituitary-adrenal (HPA) axis and stress-related autonomic and behavioral reactivity in these infants.

When looking at the role of epigenetic factors during critical developmental periods, it also is important to note that epigenetic mechanisms can act to influence the epigenome and gene expression not only during early developmental periods but throughout life. Although the organism is particularly vulnerable to environmental influences on the genome and on gene expression during periods of rapid growth and development, environmental events may influence the epigenome later in life as well. Thus, positive environmental factors, such as a healthy diet and lifestyle, can positively influence physiological and behavioral function over the life course and may possibly even rescue adverse effects of prenatal alcohol exposure or other early-life insults. Conversely, diet and other lifestyle factors may adversely influence the epigenome and possibly alter gene expression over time, as indicated by the discussion above on the effects of adult alcohol intake on the epigenome.

### Effects of Prenatal Alcohol Exposure on the Methionine–Homocysteine Cycle

As mentioned above, the methionine–homosysteine cycle is a target for alcohol’s actions and mediates some of its adverse effects. Important work by the research group led by Jennifer Thomas (Ryan et al. 2008; Thomas et al. 2004, 2009) has focused on the effects of choline—a nutrient essential for brain and behavioral development and a key component of the methionine–homocysteine cycle—on alcohol’s teratogenic effects. As discussed above, this cycle involves conversion of methionine to SAM with the subsequent generation of homocysteine and the regeneration of methionine through folate-dependent or folate-independent methylation of homocysteine. Of note, the folate-independent pathway involves the contribution of methyl groups from choline. Using well-established animal models involving either prenatal alcohol exposure or early postnatal exposure (which would be equivalent to third-trimester exposure in humans), these investigators have demonstrated that choline supplementation during the period of alcohol exposure may reduce the severity of several fetal alcohol effects, including reductions in birth and brain weights, delays in eye opening and incisor eruption, and impairments in performance on learning and memory tasks. Given the importance of choline in the methione–homocysteine cycle, and the role of this cycle in generating methyl groups for reactions central to the creation of epigenetic marks, it is tempting to speculate that the protective effects of choline are at least in part mediated by epigenetic mechanisms.

The methionine cycle also is a focus of research in a collaborative project among several laboratories (J. Weinberg, S. Innis and A. Devlin, University of British Columbia) that is examining possible epigenetic mechanisms involved in the adverse effects of prenatal alcohol exposure, and in particular, in fetal programming of the offspring stress system. Welberg and Seckl (2001) have suggested that the resetting of key hormonal systems by early environmental events may be one mechanism linking early life experiences with long-term health consequences. Importantly, the HPA or stress system appears to be highly susceptible to programming during development (Matthews 2000; Matthews et al. 2002; Phillips et al. 1998). Studies both in children with FASD and in animal models of prenatal alcohol exposure have shown that alcohol exposure in utero programs the fetal HPA axis such that the HPA system shows greater reactivity or responsiveness throughout life (for reviews, see Hellemans et al. 2010; Sliwowska et al. 2006; Weinberg et al. 2008; Zhang et al. 2005). Studies in rodents currently are investigating the possibility that HPA programming by in utero alcohol exposure may be mediated by alterations in the methionine–homocysteine cycle that subsequently result in changes in DNA methylation and gene expression patterns. Preliminary findings to date support this suggestion, indicating increased plasma levels of methionine and alterations in the regulating enzymes both in the alcohol-consuming pregnant female and in the fetus (O’Neill et al. 2007; Sulistyoningrum et al. 2010).

## Conclusions and Outlook

From the discussion above, it is apparent that future studies focused on both genetic and epigenetic mechanisms and, importantly, on potential links between the two, likely will help to illuminate key mechanisms through which alcohol interacts with critical target pathways to reprogram genes. Studies focused on genetic variation in the gene encoding an enzyme called methylenetetrahydrofolate reductase (MTHFR), which is a key enzyme in the methionine–homocysteine cycle, are a good example of this type of research (e.g., Lutz 2008). For example, a genetic variant in the MTHFR gene, called *MTHFR C677T*, results in higher plasma homocysteine levels, an effect similar to that seen during chronic alcohol intake. Because homocysteine is involved in the metabolism of methyl groups used for DNA and histone methylation, it is likely that such variants ultimately will affect the epigenetic regulation of gene expression. An additional exciting layer of complexity pointing to the homocysteine pathway as being particularly sensitive to environmentally altered gene activity was uncovered by a recent report indicating that the *MTHFR* gene itself is under epigenetic control through smoking-induced DNA methylation of its promoter regions (Vaissière et al., 2009). It will be interesting to determine whether prenatal alcohol exposure exerts a similar lasting effect on *MTHFR* gene regulation or on other key genes of pathways implicated in the development of FASD.

Finally, it will be important for future studies aimed at understanding FASD to integrate genetic and epigenetic analyses closely with physiological studies. This is illustrated by a recent report suggesting that altered DNA methylation of the regulatory region of a gene encoding the enzyme monoamine oxidase A (MAOA) in white blood cells known as lymphoblasts is associated with alcohol dependence in female but not in male subjects (Philibert et al. 2008). In addition, the degree of DNA methylation in the MAOA regulatory region was somewhat correlated with genetic variation in a variable DNA sequence (i.e., nucleotide repeat) located in that region in female subjects. Future studies focusing on the close integration of genetics and epigenetics with physiological and environmental variables undoubtedly will move this field forward in significant ways and point the way to possible targeted interventions and/or treatments to attenuate the marked adverse effects of prenatal alcohol exposure.

## Footnotes

1 DNA consists of a sequence of building blocks called nucleotides. Each nucleotide comprises a base, a sugar molecule (deoxyribose), and a phosphate group. There are four bases: cytosine, guanine, thymine, and adenine.

2 Alcohol exposure in these experiments lasted from several months to approximately 3 years.

3 The alcohol concentration used was 6 *μ*/ml of 95 percent ethanol, corresponding to a final concentration of 400 mg/dl.

4 The stressor used here was the “still face” procedure, in which the mother briefly stops responding to her infant.
